# Correction to: Long Covid—a cause of concern for surgical training

**DOI:** 10.1093/jhps/hnae023

**Published:** 2024-06-29

This is a correction to: Richard E Field, Long Covid—a cause of concern for surgical training, *Journal of Hip Preservation Surgery*, Volume 9, Issue 3, August 2022, Pages 143–144, https://doi.org/10.1093/jhps/hnac039.

The following changes have been made to the originally published manuscript.

The section **FUNDING** and contents have been removed from the article.

